# Identification of potential biomarkers for diabetic cardiomyopathy using LC-MS-based metabolomics

**DOI:** 10.1530/EC-23-0384

**Published:** 2024-01-25

**Authors:** Run-Qing Xiong, Yan-Ping Li, Lu-Ping Lin, Jeng-Yuan Yao

**Affiliations:** 1 Department of Ultrasonic Imaging, Xiamen Medical College Affiliated Second Hospital, Fujian, China; 2 Key Laboratory of Functional and Clinical Translational Medicine, Fujian Province University, Xiamen Medical College, Fujian, China; 3 Department of Endocrinology, Xiamen Medical College Affiliated Second Hospital, Fujian, China

**Keywords:** diabetic cardiomyopathy, type 2 diabetes mellitus, metabolomics, biomarkers, liquid chromatography–mass spectrometry, plasma metabolite profiling

## Abstract

Diabetic cardiomyopathy (DCM) is a serious complication of type 2 diabetes mellitus (T2DM) that contributes to cardiovascular morbidity and mortality. However, the metabolic alterations and specific biomarkers associated with DCM in T2DM remain unclear. In this study, we conducted a comprehensive metabolomic analysis using liquid chromatography–mass spectrometry (LC-MS) to investigate the plasma metabolite profiles of T2DM patients with and without DCM. We identified significant differences in metabolite levels between the groups, highlighting the dysregulation of various metabolic pathways, including starch and sucrose metabolism, steroid hormone biosynthesis, tryptophan metabolism, purine metabolism, and pyrimidine metabolism. Although several metabolites showed altered abundance in DCM, they also shared characteristics of DCM and T2DM rather than specific to DCM. Additionally, through biomarker analyses, we identified potential biomarkers for DCM, such as cytidine triphosphate, 11-ketoetiocholanolone, saccharopine, nervonic acid, and erucic acid. These biomarkers demonstrated distinct patterns and associations with metabolic pathways related to DCM. Our findings provide insights into the metabolic changes associated with DCM in T2DM patients and highlight potential biomarkers for further validation and clinical application. Further research is needed to elucidate the underlying mechanisms and validate the diagnostic and prognostic value of these biomarkers in larger cohorts.

## Introduction

Diabetes mellitus, particularly type 2 diabetes mellitus (T2DM), is a global health issue affecting millions of individuals worldwide, with its prevalence continually escalating (1). T2DM is associated with a multitude of complications, including cardiovascular diseases, neuropathy, nephropathy, and retinopathy, further exacerbating the burden on public health (2). Among these, diabetic cardiomyopathy (DCM), characterized by ventricular dysfunction and heart failure independent of coronary artery disease and hypertension, has been increasingly recognized as a significant diabetic complication (3). Despite intensive research, the pathophysiology of DCM remains complex and poorly understood, involving multifactorial processes such as metabolic disturbances, myocardial fibrosis, and inflammation (4). The early detection and treatment of DCM are challenging due to the lack of specific symptoms and effective biomarkers (5). Although several biochemical and imaging markers are employed in clinical practice, their sensitivity and specificity in diagnosing DCM are not satisfactory (6, 7, 8). Hence, there is an urgent need to identify new and reliable biomarkers for DCM to improve its early detection and intervention.

Significant efforts have been made to elucidate the complex pathophysiological mechanisms underlying DCM, and numerous studies have highlighted the importance of metabolic derangements in its development and progression (9, 10). Imbalances in metabolic pathways, such as lipid metabolism, glucose metabolism, and amino acid metabolism, have been linked to the onset of DCM (11). Meanwhile, several studies have identified potential biomarkers for DCM, including blood-based markers, imaging markers, and even genetic markers, providing valuable insights into the disease's pathology (12, 13, 14).

In recent years, metabolomics, an emerging field of ‘omics’ research, has shown great promise in identifying novel biomarkers for various diseases, including DCM (15). Metabolomics provides a comprehensive analysis of small molecule metabolites in a biological sample, offering the potential to uncover novel pathways implicated in disease pathogenesis (16, 17). There have been a few studies utilizing metabolomics to explore heart failure, revealing unique metabolic profiles associated with the disease (18, 19). In the context of DCM, several metabolites have been identified using metabolomics, implicating various metabolic pathways in the pathogenesis of the disease (20, 21). However, the metabolomic landscape of DCM is still far from fully elucidated, and further investigations are warranted to confirm these findings and discover new potential biomarkers.

In this study, we employed an LC-MS-based metabolomics approach to investigate the plasma metabolite profile of T2DM patients with and without DCM. We aim to identify novel plasma biomarkers for DCM in T2DM patients and shed light on the metabolic alterations associated with this complication.

## Materials and methods

### Study design and participant information

This study was conducted at the Second Affiliated Hospital of Xiamen Medical College, Xiamen, China. Participants were recruited in May 2022. A total of 43 volunteers were recruited for the study and classified into three groups based on their clinical profiles. Table 1 provides a detailed summary of the average age, gender distribution, and key clinical parameters, such as blood glucose, cholesterol, and triglyceride concentrations, among the three groups. The table elucidates significant differences in age between the normal group and the T2DM/DCM groups, while gender distribution, cholesterol concentrations, and blood glucose levels exhibit distinct patterns across the cohorts. The normal group was participants without a history of diabetes. The T2DM group was individuals diagnosed with type 2 diabetes mellitus, but demonstrated normal cardiac function with no signs of cardiomyopathy. The DCM group was participants diagnosed with type 2 diabetes mellitus and concurrent cardiomyopathy. Primary symptoms exhibited by these individuals included reduced left ventricular diastolic function, high voltage in sinus rhythm left ventricle, ST-segment changes in some leads, T-wave changes in some leads, and low voltage in the left chest lead. The purpose of this study was to examine the distinct metabolite profiles of plasma among these groups using liquid chromatography–mass spectrometry (LC-MS) to identify potential biomarkers and metabolic pathways associated with diabetic cardiomyopathy in patients with type 2 diabetes. This study was approved by the Medical Ethics Committee of Xiamen Medical College (Approval number 20211207008). All procedures performed in studies involving human participants were in accordance with the Ethical Standards of the Institutional and/or National Research Committee and with the 1964 Helsinki Declaration and its later amendments or comparable ethical standards. Informed consent was obtained from all individual participants included in the study. The authors did not have access to information that could identify individual participants during or after data collection.

**Table 1:** Demographic and clinical characteristics of the three study groups. The data include the number of participants (*n*); the gender distribution (male/female); age; levels of blood glucose, cholesterol, and triglycerides; diabetes history; and signs of cardiomyopathy.

Groups	*n*	Male/female	Age	Blood glucose (mM)	Cholesterol (mM)	Triglyceride (mM)	Diabetes history	Signs of cardiomyopathy
Normal	16	8/8	46.3 ± 8.7^c^	5.1 ± 0.3^a^	5.0 ± 0.5	1.3 ± 0.6^b^	None	None
T2DM	12	6/6	48.5 ± 9.3	8.2 ± 2.8	4.6 ± 0.6	1.7 ± 1.0	Type 2	None
DCM	15	9/6	52.0 ± 6.7	8.3 ± 2.0	3.7 ± 1.0^a^	2.0 ± 0.9	Type 2	Reduced left ventricular diastolic function, high voltage in sinus rhythm left ventricle, ST-segment changes in some leads, T-wave changes in some leads, and low voltage in the left chest lead.

Statistical analysis was conducted using the *t*-test.

^a^Significant differences compared to the other two groups with *P* < 0.01; ^b^Significant differences compared to the DCM group with *P* < 0.05; ^c^Significant differences compared to the DCM group with *P* < 0.01.

### Sample collection

Blood samples were collected into heparinized anticoagulant tubes from all participants. To obtain plasma, the blood samples were immediately centrifuged at 1100 ***g*** for 10 min at 4°C. From the resulting plasma, 100 μL were extracted and mixed with 400 μL of methanol to ensure homogeneity. The mixture was then disrupted using an ultrasonic disruptor, followed by centrifugation at 10,000 ***g*** for 10 min at 4°C to separate the supernatant. The supernatant was collected and dried using a nitrogen blow dryer. The dried samples were then reconstituted in 100 μL of a solution of acetonitrile (ACN) and water mixed in a 1:1 ratio. After reconstitution, samples were centrifuged again at 10,000 ***g*** for 10 min at 4°C, and the supernatant was collected for metabolomic analysis. All samples were stored at −20°C until ready for use.

### Metabolomic analysis

Metabolomic analysis was performed using a Waters Xevo G2-XS QToF mass spectrometer. Chromatographic separation was conducted using a 1.7 µm ACQUITY UPLC BEH C18 column. The injection volume was set at 2 μL, with a flow rate of 0.4 mL/min. The mobile phase consisted of 0.1% formic acid in water (phase A) and ACN with 0.1% formic acid (phase B). A 30-min gradient was applied as follows: 90% A (0–2 min), a linear gradient from 90% A to 0% A (2–20 min), maintaining at 0% A (20–25 min), a swift change from 0% A to 90% A (25–26 min), and then maintaining at 90% A (26–30 min). For the mass spectrometry conditions, the ESI source was operated in positive ion mode. The capillary voltage was set at 3125 V. Cone gas flow was at 60 L/H, desolvation temperature was set at 500°C, and the source temperature was maintained at 100°C. Desolvation gas flow was set at 600 L/h. The mass spectrometer was set to scan over the range of 50–1200 m/z, and data were collected in centroid mode. Raw data were collected using Masslynx 4.1 software. To ensure the mass accuracy during data acquisition, an independent lock-mass ion, leucine enkephalin ([M+H]^+^ = 556.2771), was used.

### Data processing and metabolite identification

Raw data collected from the LC-MS were analyzed using Progenesis QI software. This software was used to perform alignment and peak picking for the data. Metabolites were selected based on a fold change greater than 2.0 and a *P*-value less than 0.05 (determined by ANOVA), which signified statistically significant changes in metabolite levels between the different groups (Supplementary Data 1, see section on supplementary materials given at the end of this article). Metabolite identification was performed by comparing the mass spectra of the selected peaks with the endogenous metabolites database of the Human Metabolome Database (HMDB). Both primary (exact mass) and secondary (MS/MS fragmentation pattern) spectral data were used for the identification process (Supplementary Data 2 and 3).

### Statistical and pathway analysis

Statistical analysis was performed using the online platform MetaboAnalyst. The data filtering was based on the interquartile range, with 40% of the data filtered out. Normalization was performed using the auto-scaling method, which scales the data so that the variance of each metabolite is equal. Significantly different metabolites in the DCM group were identified using a one-way ANOVA test. A *P*-value of less than 0.05 was considered statistically significant. Subsequently, pathway analysis was performed using the Kyoto Encyclopedia of Genes and Genomes (KEGG) database.

## Results

### Metabolomic analysis of plasma samples from DCM patients

**Figure 1:**
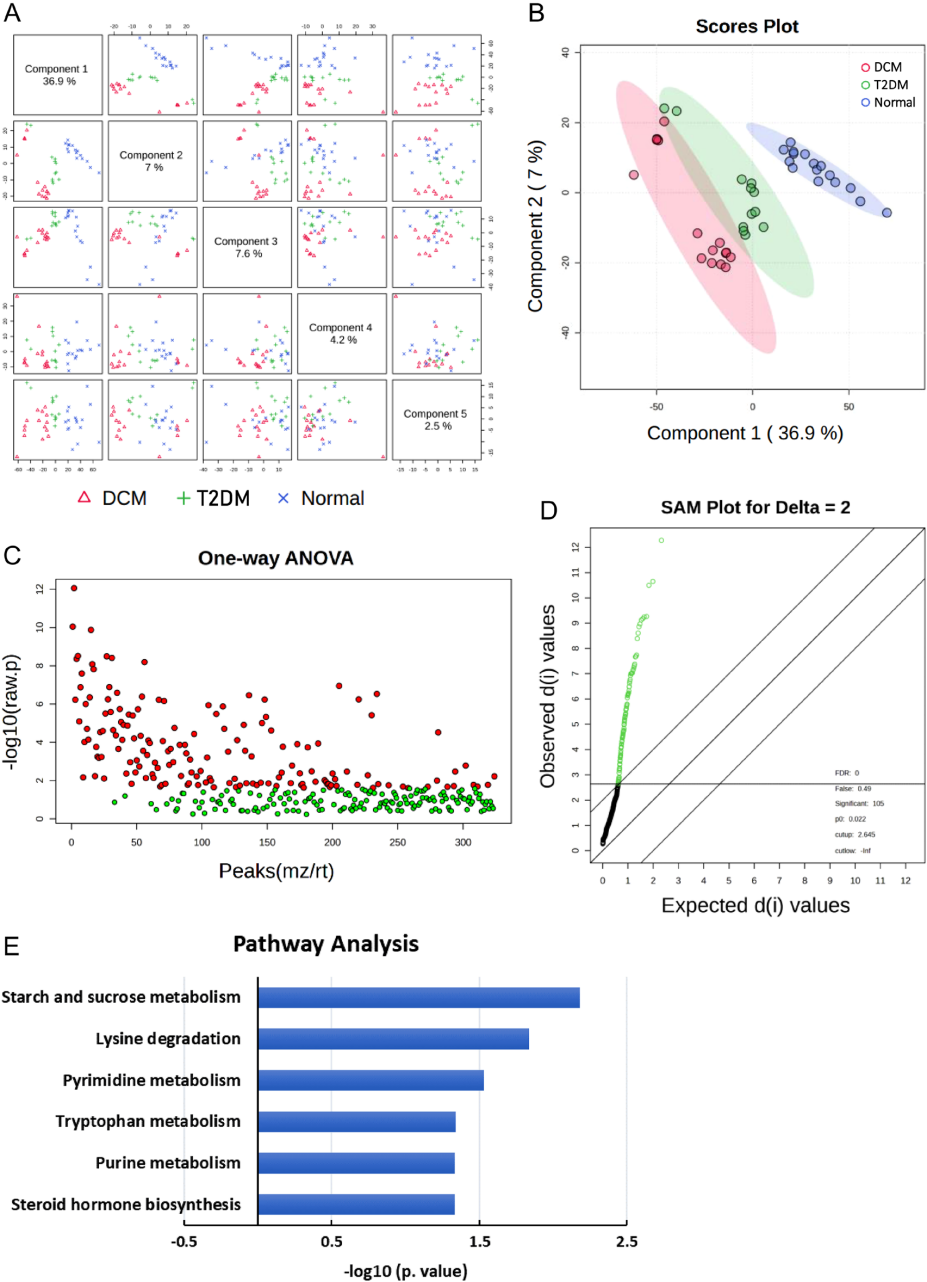
Metabolomic analysis of plasma samples from DCM group. (A) Pairwise score plot for the top five components obtained from PLS-DA analysis, demonstrating distinct clustering of normal, T2DM, and DCM groups. (B) 2D scores plot comparing components 1 and 2, further illustrating the separation among normal, T2DM, and DCM groups. (C) Data filtering and one-way ANOVA analysis identified 160 significant metabolites (red dots) and 164 insignificant metabolites (green dots) among the 361 endogenous metabolites. (D) Significance analysis of metabolomics revealed 105 metabolites exceeding the specified threshold as significant features. (E) Pathway analysis of the significant metabolites. The analysis identified significantly altered metabolic pathways (*P* < 0.05) in DCM plasma.

The metabolomic analysis of plasma samples using LC-MS and subsequent data processing yielded a total of 27,143 ions. Among these ions, 4771 exhibited statistically significant differences (*P* < 0.05, ANOVA) and fold changes greater than 2 among the three study groups (normal, T2DM, and DCM). To further investigate the metabolic differences between the groups, the 4771 ions were subjected to analysis using MetaboAnalyst 5.0. A partial least squares-discriminant analysis (PLS-DA) was performed, and pairwise score plots for the top five components were generated (Fig. 1A). The 2D scores plot comparing components 1 and 2 (Fig. 1B) clearly demonstrated distinct separation among the normal, T2DM, and DCM groups, with a significant difference observed between the normal group and the other two groups. The 4771 ions were also compared against the HMDB using Progenesis QI software, resulting in the identification of 361 endogenous metabolites.

After data filtering in MetaboAnalyst 5.0, the remaining 361 metabolites were subjected to one-way ANOVA and *post hoc* tests, with a *P*-value (false discovery rate (FDR)) cutoff of 0.05. This analysis revealed 160 significant metabolites (red dots) and 164 insignificant metabolites (green dots) (Fig. 1C). Additionally, a significance analysis of metabolomics was performed, with a delta value (FDR control) set at 2 (Fig. 1D).

In Fig. 1E, we performed pthway analysis using the significant metabolites identified in Fig. 1C. This analysis was conducted using the KEGG metabolic pathways as the backend knowledgebase. The results revealed several significantly altered metabolic pathways (*P* < 0.05) in the plasma of DCM patients. The metabolic pathways that showed significant differences in DCM plasma included steroid hormone biosynthesis, purine metabolism, tryptophan metabolism, pyrimidine metabolism, lysine degradation, and starch and sucrose metabolism.

### Metabolites with significant abundance changes in DCM group

Based on the results of the one-way ANOVA, metabolites with an *F* value > 4, a *P* value, FDR < 0.05, and an abundance greater than 1000 in the mass spectrometry data were selected. In the DCM group, the metabolites with the highest abundances were identified as 3-hydroxytetradecanedioic acid, 17α-ethynylestradiol, phytanic acid, erucic acid, nervonic acid, methylguanosine, 19,20-DiHDPA, thromboxane B3, dCTP, N8-acetylspermidine, and 11-β-hydroxyandrosterone-3-glucuronide. On the other hand, the metabolites with the lowest abundances in the DCM group were identified as CTP, glucose 6-phosphate, saccharopine, uridine diphosphate glucose, 11-ketoetiocholanolone, inosinic acid, 3-methylcrotonylglycine, aldosterone, and docosapentaenoic acid (Table 2).

**Table 2:** Metabolites with significant abundance changes in DCM group. This table presents the metabolites showing significant changes in abundance in the plasma of individuals with DCM compared to the normal and T2DM groups. The metabolites were selected based on their *F* value (>4), *P* value, and false discovery rate (FDR) (<0.05) from the results of one-way ANOVA analysis. Only metabolites with an abundance greater than 1000 were included in the analysis.

Description	*F* value	*P*	FDR	Compound ID	Adducts	m/z	DCM trend	Relative abundance		
								DCM	T2DM	Normal
Aldosterone	33.18	3.2E-09	2.01E-07	HMDB0000037	M + H	361.194	↓	0.3 ± 0.20	0.3 ± 0.33	1.0 ± 0.28
Docosapentaenoic acid	24.19	1.3E-07	3.08E-06	HMDB0001976	M + H	331.263	↓	0.5 ± 0.27	0.5 ± 0.15	1.0 ± 0.23
N8-Acetylspermidine	22.70	2.58E-07	5.58E-06	HMDB0002189	M + ACN + H	229.204	↑	2.6 ± 0.72	2.2 ± 0.66	1.0 ± 0.63
Inosinic acid	22.42	2.95E-07	5.97E-06	HMDB0000175	M + H	349.052	↓	0.4 ± 0.31	0.5 ± 0.24	1.0 ± 0.25
CTP	20.63	6.97E-07	9.03E-06	HMDB0000082	M + H	483.995	↓	0.3 ± 017	0.5 ± 0.20	1.0 ± 0.47
Glucose 6-phosphate	19.32	1.34E-06	1.55E-05	HMDB0001401	M + ACN + H	302.061	↓	0.4 ± 0.24	0.5 ± 0.21	1.0 ± 0.40
Saccharopine	18.68	1.86E-06	2.01E-05	HMDB0000279	M + ACN + H	318.167	↑	2.5 ± 0.91	1.5 ± 0.66	1.0 ± 0.52
Uridine diphosphate glucose	17.32	3.81E-06	3.53E-05	HMDB0000286	M + ACN + H	608.079	↓	0.5 ± 0.36	0.6 ± 0.29	1.0 ± 0.17
11-Ketoetiocholanolone	14.56	1.77E-05	0.0001	HMDB0006031	M + ACN + H	346.235	↓	0.2 ± 0.13	0.4 ± 0.24	1.0 ± 0.68
3-Methylcrotonylglycine	13.63	3.06E-05	0.0002	HMDB0000459	M + ACN + H	199.110	↓	0.4 ± 0.29	0.5 ± 0.31	1.0 ± 0.37
17α-Ethynylestradiol	9.65	0.0004	0.0016	HMDB0001926	M + ACN + H	338.215	↑	2.2 ± 0.99	1.5 ± 0.60	1.0 ± 0.64
Phytanic acid	9.02	0.0006	0.0024	HMDB0000801	M + H	313.310	↑	4.2 ± 3.02	2.7 ± 1.95	1.0 ± 0.37
Erucic acid	8.03	0.0012	0.0043	HMDB0002068	M + H	339.325	↑	2.3 ± 1.21	1.5 ± 1.01	1.0 ± 0.28
Nervonic acid	7.66	0.0015	0.0056	HMDB0002368	M + H	367.352	↑	2.2 ± 1.08	1.4 ± 1.06	1.0 ± 0.32
Methylguanosine	5.89	0.0057	0.0177	HMDB0001563	M + ACN + H	339.144	↑	5.2 ± 4.88	3.2 ± 3.27	1.0 ± 0.73
11-β-Hydroxyandrosterone-3-glucuronide	5.00	0.0116	0.0310	HMDB0010351	M + ACN + H	524.283	↑	2.2 ± 1.55	1.3 ± 0.87	1.0 ± 0.30
19,20-DiHDPA	4.80	0.0136	0.0354	HMDB0010214	M + H	363.246	↑	2.4 ± 1.97	1.7 ± 0.74	1.0 ± 0.55
Thromboxane B3	4.77	0.0139	0.0354	HMDB0005099	M + H	369.233	↑	3.5 ± 3.71	1.7 ± 1.47	1.0 ± 0.16
dCTP	4.44	0.0182	0.0403	HMDB0000998	M + H	468.000	↑	11.8 ± 14.57	6.0 ± 10.13	1.0 ± 0.56
3-Hydroxytetradecanedioic acid	4.35	0.0195	0.0425	HMDB0000394	M + ACN + H	316.214	↑	2.4 ± 2.19	2.2 ± 0.99	1.0 ± 0.52

### Biomarker analyses between the T2DM and DCM groups

**Figure 2:**
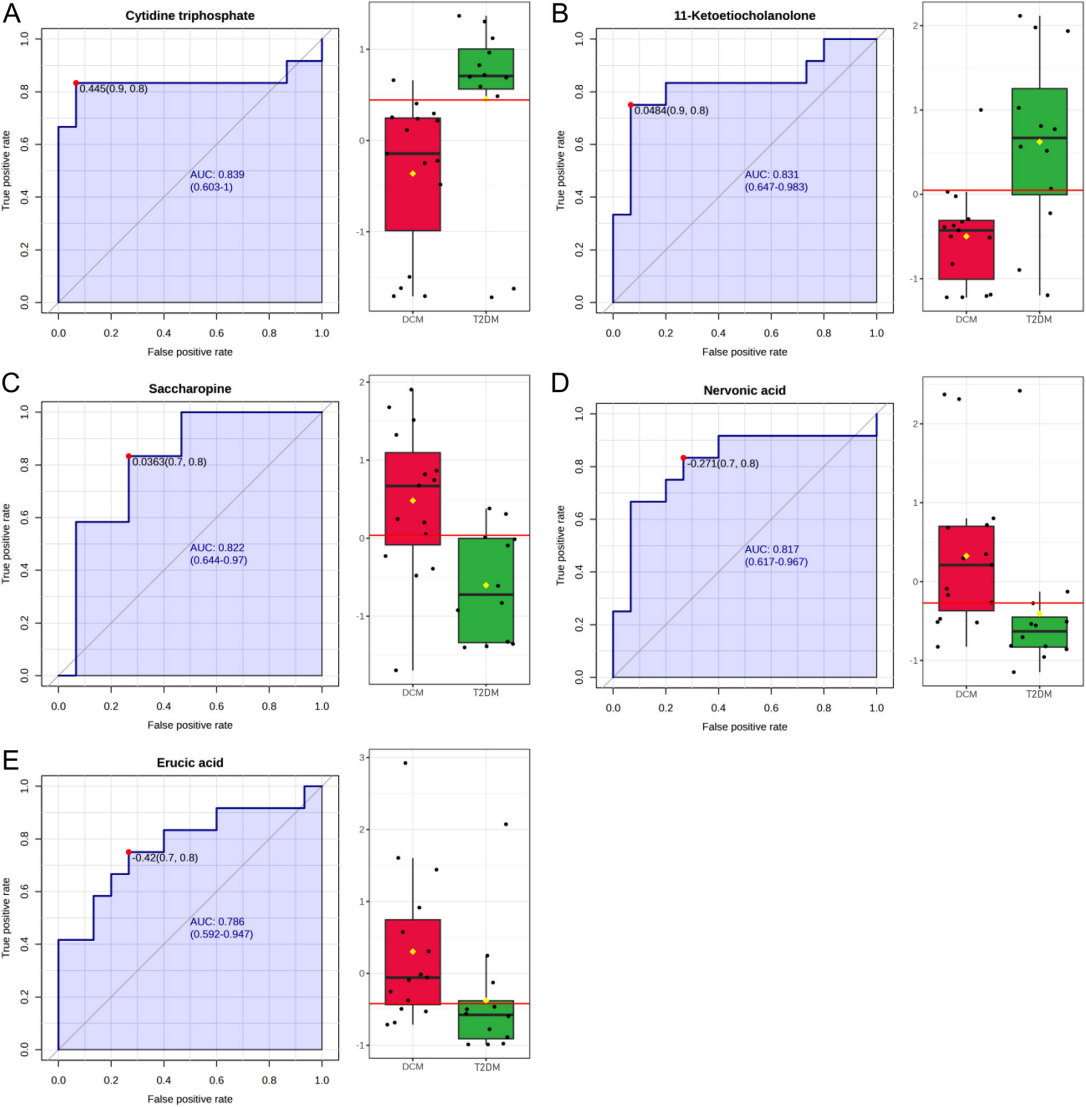
ROC curve-based biomarker analyses between the T2DM and DCM groups. (A) Cytidine triphosphate (CTP). (B) 11-Ketoetiocholanolone. (C) Saccharopine. (D) Nervonic acid. (E) Erucic acid. The left panel of each subfigure shows the ROC curve, which assesses the performance of the individual biomarker. The sensitivity is on the *y*-axis, and the specificity is on the *x*-axis. The area under the curve (AUC) is in blue. The right panel displays the box plot, depicting the relative abundances of the selected feature between the T2DM and DCM groups within the dataset. A horizontal line is in red indicating the optimal cutoff.

In further analysis of the differential metabolites between the T2DM and DCM groups, we employed ROC curve-based biomarker analyses to identify specific biomarkers that could distinguish DCM from T2DM. Figure 2 presents the five metabolites with an area under the curve (AUC) greater than 0.75, which indicates their potential as specific biomarkers for DCM. The selected biomarkers and their corresponding ROC curves are as follows: CTP (Fig. 2A), 11-ketoetiocholanolone (Fig. 2B), saccharopine (Fig. 2C), nervonic acid (Fig. 2D), and erucic acid (Fig. 2E). The left panel of each figure shows the ROC curve of an individual biomarker, while the right panel displays the box plot depicting the relative abundances of the selected feature between the two groups within the dataset.

## Discussion

Our study aimed to identify potential plasma biomarkers for DCM in T2DM patients and investigate the associated metabolic alterations. Although we identified several compounds that exhibited the highest or lowest levels in the DCM group compared to the normal group (Table 2), most of these compounds were shared characteristics between DCM and T2DM, rather than unique to DCM. For example, we observed lower levels of aldosterone, docosapentaenoic acid, inosinic acid, glucose 6-phosphate, and uridine diphosphate glucose in both the DCM and T2DM groups compared to the normal group. Conversely, we found higher levels of N8-acetylspermidine, 3-hydroxytetradecanedioic acid, 17α-ethynylestradiol, phytanic acid, methylguanosine, 11-β-hydroxyandrosterone-3-glucuronide, 19,20-DiHDPA, thromboxane B3, and dCTP in both the DCM and T2DM groups (Table 2). These metabolites may be associated with the progression of diabetes; however, they are not sufficient to serve as specific markers for DCM in our statistical model. The altered metabolites identified in our study are involved in various metabolic pathways, including starch and sucrose metabolism, steroid hormone biosynthesis, and tryptophan metabolism (Fig. 1E).

Biomarker analyses using ROC curves identified several potential biomarkers for DCM, including CTP, 11-ketoetiocholanolone, saccharopine, nervonic acid, and erucic acid (Fig. 2). Notably, the decreased level of CTP, which is associated with pyrimidine metabolism (Fig. 1E), was found in the DCM group. Additionally, the downregulation of inosinic acid (Table 2) was associated with purine metabolism pathway (Fig. 1E). Disorders of purine and pyrimidine metabolism have been extensively studied (22, 23, 24), particularly in the context of hyperuricemia and gout (25).

Saccharopine, an intermediate in the metabolism of the amino acid lysine, exhibits significantly higher levels in individuals with DCM compared to those with T2DM and normal subjects (Table 2 and Fig. 2C). The altered abundance of saccharopine is closely associated with disruptions in the lysine degradation pathway (Fig. 1E). It is noteworthy that saccharopine has been identified as a mitochondrial toxin, and its abnormal accumulation can lead to defective mitochondrial dynamics and impaired function in experimental models (26, 27, 28). The lysine degradation pathway is clinically relevant, as it has been implicated in severe neurometabolic disorders such as pyridoxine-dependent epilepsy and glutaric aciduria type 1 (29). The significant elevation of saccharopine levels in DCM suggests a potential link between lysine metabolism, mitochondrial dysfunction, and the pathogenesis of DCM.

11-Ketoetiocholanolone, a metabolite of cortisol, has been widely employed as a stress biomarker in various vertebrate species (30, 31). Although its precise physiological significance remains unclear, our findings suggest that decreased levels of 11-ketoetiocholanolone may serve as a potential marker for DCM. The observed reduction in 11-ketoetiocholanolone levels in DCM patients implies alterations in the cortisol metabolism pathway or stress response mechanisms associated with the development and progression of DCM.

Nervonic acid is a very long-chain fatty acid primarily found in mammalian nerve tissues. Its significance extends beyond its structural role, as emerging research has identified its potential as a biomarker for various disorders. For instance, studies have indicated that plasma nervonic acid levels hold promise as a potential biomarker for major depressive disorder. Furthermore, the presence of nervonic acid has been associated with important physiological processes, including myelination and neuroinflammation (32, 33).

The presence of erucic acid, a fatty acid, has been associated with the inhibition of mitochondrial oxidation of other fatty acids, particularly in cardiac tissues. This inhibition can lead to the accumulation of triglycerides in the hearts of rats fed rapeseed oil (34, 35). These findings suggest that erucic acid and its mitochondrial metabolites play a significant role in modulating lipid metabolism and cardiac function. In the context of DCM, further investigation is necessary to explore the potential involvement of erucic acid and its metabolites in the pathogenesis of DCM. Understanding the impact of erucic acid on cardiac lipid metabolism may contribute to the development of novel therapeutic strategies targeting lipid dysregulation in DCM.

In conclusion, our study identified potential plasma biomarkers and metabolic alterations associated with DCM in T2DM patients. The findings contribute to the understanding of the complex metabolic changes in DCM and highlight the need for further research to validate these biomarkers and explore their clinical applications. Continued efforts in elucidating the metabolic pathways and mechanisms involved in DCM may ultimately lead to improved early detection, risk stratification, and therapeutic strategies for this debilitating complication of T2DM.

## Supplementary materials

Supplementary Data 1: Peaks of All Compounds. This dataset offers a comprehensive insight into 4774 ions observed across individual samples, highlighting statistically significant differences (p < 0.05, ANOVA) and fold changes greater than 2 among the three study groups (Normal, T2DM, and DCM). It includes essential details such as m/z, Retention time (min), Chromatographic peak width (min), Anova (p), q Value, Normalised abundance, and other relevant parameters for each identified ion.

Supplementary Data 2: Peaks of Identified Endogenous Metabolites. This dataset provides information on 361 endogenous metabolites identified among 4,771 ions compared against the Human Metabolome Database (HMDB) using Progenesis QI software. The details include m/z, Retention time (min), Chromatographic peak width (min), Anova (p), q Value, Normalised abundance, and other pertinent parameters for each identified metabolite.

Supplementary Data 3: Potential Identifications of 361 Endogenous Metabolites. This dataset presents potential identifications for 361 endogenous metabolites, encompassing Human Metabolome Database (HMDB) ID, Formula, Score, Mass Error (ppm), Isotope Similarity, m/z, Retention time (min), and other pertinent identification parameters for each metabolite.

## Declaration of interest

The authors declare that there is no conflict of interest that could be perceived as prejudicing the impartiality of the study reported.

## Funding

This work was supported by the Program for New Century Excellent Talents in Fujian Province University (NC2018-47), Natural Science Foundation of Fujian Provincehttp://dx.doi.org/10.13039/501100003392, China (2022D024).

## Data availability

All relevant data are within the paper and its supporting information files.
